# Stakeholder views of podiatry services in the UK for people living with arthritis: a qualitative study

**DOI:** 10.1186/s13047-020-00427-7

**Published:** 2020-09-24

**Authors:** Charlotte Dando, Dawn Bacon, Alan Borthwick, Catherine Bowen

**Affiliations:** 1 School of Health Sciences, Faculty of Environmental and Life Sciences, University of Southampton, Highfield Campus Building 67, University Road, Southampton, Hampshire SO17 1BJ UK; 2 The Academy of Research and Improvement, Solent NHS Trust, Southampton, UK; 3 Centre for Sport, Exercise and Osteoarthritis Research Versus Arthritis, Southampton, UK

**Keywords:** Arthritis, Podiatry, Footcare, Service provision, Foot health, Mobility, Pain management, Independence

## Abstract

**Background:**

The aim of this study was to explore the views of stakeholders in podiatry services, patients, commissioners and general practitioners (GP), to further understand experiences of referral, access and provision of treatment in the National Health Service (NHS) for foot problems for patients living with arthritis.

**Method:**

To explore in-depth individual views and experiences of stakeholders in podiatry services, 19 patients who had arthritis (osteoarthritis and/or rheumatoid arthritis) participated in one of four focus groups. In addition, seven commissioners and/or GPs took part in semi structured interviews. A purposive sampling strategy was adopted for all focus groups and semi structured interviews. To account for geographical variations, the focus groups and semi structured interviews were conducted across two predetermined regions of the United Kingdom (UK), Yorkshire and Hampshire. Data was rendered anonymous and transcribed verbatim. Thematic analysis was employed to identify key meanings and report patterns within the data.

**Results:**

Five key themes derived from the focus groups and interviews suggest a variety of factors influencing referral, access and provision of treatment for foot problems within the UK. 1. Systems working together (navigation of different care pathways, access and referral opportunities for people with OA or RA, education around foot health services for people with OA or RA); 2.Finance (financial variations, different care systems, wasting resources); 3. Understanding what podiatry services have to offer (podiatrists are leaders in foot health services, service requirements in relation to training standards and health needs); 4. Person factors of foot pain (arthritis is invisible, affects quality of life, physical and mental wellbeing); 5. Facilitators of foot care (NICE guidelines, stakeholder events, supporting self-management strategies).

**Conclusion:**

The findings indicate that patients, commissioners and GPs have very similar experiences of referral, access and provision of treatment for foot problems, for patients living with arthritis. Essentially, commissioners and GPs interviewed called for a transformational approach in current systems to include newer models of care that meet the footcare needs of individual patient circumstances. Patients interviewed called for better signposting and information about the different services available to help them manage their foot health needs. To address this, we have formulated a signposting pack for all stakeholders to help them facilitate access to appropriate clinicians ‘at the right time, in the right place’ to manage foot health problems.

## Background

Physical activity, including walking is promoted as a strategy to maintain healthy lifestyles, prevent health problems and minimise the impact of disease processes [1, 2]. However for some of the twenty million people in the UK living with arthritis the presence of debilitating foot pain, which has been linked to falling, tripping, weight gain and social isolation [3, 4], means that they either continue to walk despite foot pain, in a bid to gain health benefits; or that they reduce their walking activity levels.

Within the UK, GPs are the first point of contact for most NHS patients; their referral and gate-keeping roles have been accepted practice for over a century [5–9]. Foot pain encounters by UK GPs are not insubstantial. In a previous study using the UK National Institute for Health Research (NIHR) Clinical Practice Research Datalink (CPRD) reports of foot and ankle pain encounters, we showed that over the four-year study period 346,067 patients reported foot and/or ankle pain; amounting to 1.8% of all GP encounters [10]. The GP’s recorded the encounter of foot and ankle pain at the patient’s appointment. We also identified that, on average, people who have foot or ankle pain are likely to see their GP more than once for this problem [10]. There appears to be a similar pattern reported in Australia for GP encounters relative to foot and ankle osteoarthritis (OA) [11] and management of hallux valgus [12]. Despite this notable burden of foot problems relative to musculoskeletal conditions, an unmet need for their management has been consistently reported within the UK [13–18] and a lack of appreciation of foot care needs within GP consultations in the UK is suggested as a key concern for these patients [19].

Notably we found that the majority of people with foot pain are referred to orthopaedics and physiotherapy, with fewer than half being referred to podiatry [10]. Menz et al. (2019) suggest that foot problems such as hallux valgus may not be considered by GPs as chronic conditions and that GPs primarily use the term chronic conditions for referring patients to podiatrists for the management of foot problems related to diabetes [12]. To our knowledge, the only large investigations are focused on OA and hallux valgus. There appears to be a focus on hallux valgus in contrast with limited literature on rheumatoid arthritis (RA), though people with RA are core to data generation Echoing these findings, a systematic review of current NHS guidelines, standards of care and recommendations for people with chronic conditions found that the literature on foot-care and/or podiatry is also concentrated around the assessment and prevention of foot and ankle problems related to complications of diabetes [20].

Uncertainty about ‘patient access criteria’ for UK NHS health care in relation to foot problems has also been reported [21] and perceived confusion over the definition of podiatry, podiatrists’ skill set and where podiatrists can be of most benefit within the UK NHS organisation [21] compounds the situation. These uncertainties are situated in the aftermath of the global financial crisis - which within the UK prompted a discourse of a sustainable national economy as the primary goal and which gave rise to fiscal constraints that came to be accepted as a matter of necessity [22, 23].

The aim of this study was to explore the experiences of stakeholders (patients, commissioners of NHS services and general practitioners) of referral, access and provision of treatment in the NHS for foot and ankle problems for people living with arthritis.

## Methods

This study forms Phase 2 of a larger body of work (the OptiFooT study) to inform the development of an optimal foot care package for individuals who have arthritis (https://www.fundingawards.nihr.ac.uk/award/CDF-2015-08-032). Phase 1 involved a systematic review to understand extant recommendations for foot-care for people with arthritis [20]; analysis of data from the UK GP clinical-practice-research-datalink (CPRD) on foot-care referral patterns for people with arthritis [10]; and exploration of UK podiatry clinicians’ views of the current provision of foot care for individuals who have arthritis [21].

Phase 2 follows on, exploring the perceptions of stakeholders (GPs, NHS commissioners and patients) on the provision of foot care in the UK for patients with arthritis. A qualitative methodology, featuring semi-structured in-depth and focus group interviews was used to capture the perspectives of stakeholders on their experiences of referral, access and treatment provision for people with arthritis related foot problems [24, 25].

Following ethical committee approval (IRAS: 15/SW/0251), data was collected from individuals who had OA or RA and from GPs and Commissioners of UK NHS services. Thus, triangulation of data from different stakeholder perspectives was embedded in the study design. All participants gave full written informed consent prior to data collection.

Semi structured interviews for GPs and commissioners, and focus groups for patients, were chosen as the most appropriate approach to capture a large amount of information in a relatively short period of time [26] and allowed us to not only to identify the issues that the commissioners and/or GPs raised, but also allowed for the observation of how patients discussed their issues in a ‘natural’ social setting. The methods adopted reflected existing standards for robustness in qualitative research, deploying triangulation of data and data saturation, which guided the final sample size [24, 25, 27].

### Participants

For individuals who had arthritis (OA and/or RA), letters of invitation were sent out to volunteers on the School of Health Sciences, University of Southampton volunteer database and the PPI and volunteer database of the NIHR Leeds, Biomedical Research Centre. Recruitment posters were also placed, with permission, at the Southampton General Hospital and the Adelaide Primary Care clinical site, Southampton.

For GPs, a recruiting call in the ‘Research Opportunities’ emails was sent by the Clinical Innovation and Research Centre (CIRC) of the Royal College of General Practitioners and recruitment flyers were sent to GP practices in the two UK zones. Utilising a snowball sampling strategy [24, 28] these informed respondents were asked to identify other appropriate GP participants. For commissioners, professional networks with local NHS Clinical Commissioning Groups (CCGs) and Podiatry Services were used to identify individuals to participate. CCGs were also approached to assist with recruitment.

A purposeful sampling strategy was utilised therefore, consistent with the qualitative study design adopted. To enable a ‘snapshot’ of two representative areas within England, two zones were established; Yorkshire (North England) and Hampshire (South England); focus groups and interviews were held in each of the zones. The two ‘zones’ each described a radius of 20–25 miles from a city. Each zone was mapped to incorporate several CCGs several NHS Trusts (both acute and community), a multitude of GP practices and a diverse pool of patients. This allowed recruitment without undue pressure whilst ensuring that the zones were of comparable size and composition to provide significant similarity between the two zones.

#### Inclusion criteria

All participants

- aged 18 or over
- able to give consent to participate in the study
- working within one of the 2 predefined project zones

Patient specific

- confirmed consultant diagnosis of rheumatoid arthritis (RA patient group)
- or confirmed medical diagnosis of osteoarthritis (OA patient group)
- have lived experienced of foot problems

Commissioner specific

- currently employed as an NHS Commissioner

GP specific

- employed NHS General Practitioner

#### Exclusion criteria

All participants

- unable to speak English
- unable to understand English
- aged under 18
- unable to give informed consent

Patient specific

- inflammatory arthritis diagnosis, other than rheumatoid arthritis
- unable to cognitively participate in focus group discussions

Commissioners and GP specific

- unable to cognitively participate in telephone interviews

Those interested in joining the study were emailed an information sheet, along with the contact details of the OptiFooT research assistant (LMc). Interested stakeholders then contacted the OptiFooT research assistant (LMc) for additional information, to have any further questions answered and be screened against the project criteria.

### Data collection

Qualitative data were generated using in-depth and focus group interviews guided by a semi-structured interview schedule (Dec 2017-April 2018) through a purposeful sampling technique. General topics for discussion were identified with a pre-determined interview schedule of questions written prior to the focus groups and interviews. The interview schedule was informed by, and constructed from, the findings from analysis of a systematic review of the literature relative to evidence for podiatry and foot care conducted by the research team [20]. During the study protocol develop GP and PPI were asked to review and comment upon the proposed schedules (appendix 1).

Each focus group was conducted by the OptiFooT research assistant (LMc) and the lead investigator (CB) as note-taker to aid with reflection, transcription and subsequent coding. One-to-one interviews were conducted by the OptiFooT research assistant (LMc). Due to the potential geographical spread of participants one-to-one interviews were offered either in person or by telephone. Validation of the transcripts was undertaken with the Leeds focus group. Findings were sent to participants and no changes were made. One GP also reviewed their transcript for interpretation accuracy. There was an attempt to balance recruitment numbers but recruitment in Southampton was slower than in Leeds for patients and vice versa in Leeds for GP’s/commissioners.

### Data analysis

Digital audio-recordings of interviews were transcribed verbatim, anonymised and imported into a data analysis package (N-Vivo 11). Using this and manual methods, codes were generated by noting recurring comments and used to categorise responses by the researchers (CD and CB). Using constant comparison, the codes were refined, compared and grouped into similar features which served as potential themes (CD, DB and CB).

Thematic analysis was identified as a suitable method to search for patterns related to the patients’, commissioners’ and GPs’ views on podiatry services for individuals living with arthritis [24, 25]. Emerging themes were discussed by the wider research team (CD, DB, CB) for verification, identification of any additional areas of interest and consensus via discussion of patterns across the data. Potential themes were repeatedly discussed by CD, DB, CB to identify any alternative interpretations. The process of verifying themes as a team provided a more rigorous approach, different perspectives and agreement on final themes.

## Results

The study recruited 26 stakeholder participants in total. Four focus groups were conducted with patients who had either OA or inflammatory arthritis (RA/Psoriatic Arthritis PsA) (*N* = 20) and four interviews were conducted with General Practitioners (GPs) and Commissioners (*N* = 4) and one focus group (*N* = 3). Characteristics of the participant focus groups (patients) and interviews and focus group (Commissioners and GPs) are described in Tables 1 and 2. Five key themes were constructed via thematic analysis and are presented in Table 3 with the subthemes. An abridged summary, with excerpts of data drawn from the transcripts, is presented below.

**Table 1:** Patient participant characteristics (*N* = 19)

	Focus group 1	Focus group 2	Focus group 3	Focus group 4
Number of Participants (female:male)	2:1	2:9	1:1	3:0
Location	Yorkshire	Yorkshire	Hampshire	Hampshire
Arthritis condition*	OA:3	OA:4 RA:3 PsA:2 Missing data:2	OA:2	OA:3

*OA Osteoarthitis; *RA* Rheumatoid arthritis; *PSA* Psoriatic arthritis

**Table 2:** GP & Commissioner participant characteristics (*N* = 7)

Participants	Interview 1	Interview 2	Interview 3	Interview 4	Focus Group
Number of Participants (female:male)	1 Female	1 Female	1 Female	1 Male	2 Female 1 Male
Location	Hampshire	Hampshire	Hampshire	Yorkshire	Hampshire

**Table 3:** Key themes and subthemes

Themes	Sub-themes
1. Systems working together / Navigation of care pathways	• Referral and access to podiatry/foot care for people with RA and OA • Different systems and service configurations for podiatry in the NHS: musculoskeletal and diabetes services • Commissioners as ‘brokers’ to achieve more productive collaborations • Education on what the podiatry / foot care services provide for people with RA or OA.
2. Finance	• Variations in cost effectiveness of podiatry services • Split systems of care (community v acute) • Guilt at wasting resources
3. Understanding what podiatry services have to offer	• Right person, right place at the right time • Podiatrists as leaders of foot health services • Service requirement in relation to training standards to meet care needs
4. Person factors of foot pain	• Arthritis is invisible as people do not complain • Foot pain affects quality of life, physical and mental wellbeing • Traditional clinical approaches encourage dependencies
5. Facilitators to foot care	• NICE guidelines • Stakeholder events • Increase focus on supporting self-management strategies

### Theme one (systems working together)

This theme represents the perceptions of what the health and social care services such as the NHS or private practice does offer in terms of foot care for people living with arthritis. Patients acknowledged the role of General Practitioners (GPs) in identifying what health and care needs they required and who should be able to provide this. Some patients were reflective of the GP role within the health service.

“… a GP has to be jack of all trades and master of none, really, haven’t they? Because they’ve got to be able to pick up anything from anybody and then know where to transfer them”. (Patient-code- LP1).

Other patients articulated their expectations of what a consultation with their GP about their foot symptoms and arthritis should be like, however most felt there was difficulty in accessing a person to look at their foot problem.

“It’s still the initial reluctance to get somebody to actually physically want to take your sock off and look at your foot. Because the minute you mention foot, faces glaze over”. (Patient-code- S3).

Patients did relate a desire for their foot health needs to be managed in one clinical visit, indicating the burden involved in having to see different clinicians (GPs, nurses, podiatrists) at different times with underlying frustration in the current system not meeting those needs.

“Well, we don’t really have one stop shops. We’ve got lots of specialists”. (Patient-code- LP11).

Commissioners and GPs also identified confusion over access and referral to podiatry/foot care services suggesting it was because those services fall between the two systems of health care and social care.

“*Yes, I think also having a condition that affects your mobility has a big impact on your health and wellbeing overall. So, you may then, like you mentioned be reluctant to leave your home, then you’ve lost that social engagement if you don’t... perhaps you live on your own then loneliness becomes an issue. So, and then that has another impact on social care services too. So, mobility I think and we all take our mobility completely for granted don’t we. But I think it really can have a huge impact.” (Commissioner – code-5).*

*‘I think your point about social elements of healthcare is really important, because we do see that in some services. That actually, there’s a significant social element to what we’re paying for, as it were. So, I think where health and social care has been so divided over such a long period of time; that real kind of, that’s your money. That’s our money. The whole pooling of budgets has never really worked.” (Commissioner – code- 4).*

They saw themselves as ‘brokers’ to achieve more productive collaborations. Most emphasised the importance of working together as a ‘whole systems approach’.

“Podiatrists have got the complicated stuff. I rely on them to tell me what they think is the most important things they have to do.” (Commissioner-code-1).

One commissioner praised one of their local podiatry teams who had been pro-active with them in producing a business case to provide a wider range of services beyond acute foot care for patients who have foot complications due to diabetes and being successful in securing those funds. They believed that, whilst they knew what podiatry is, they were explicit that there was a greater need for education on what the podiatry / foot care services provide for people with arthritis.

### Theme two: finance

This theme presents the perceptions of financing and evidencing podiatry / foot care services for people living with arthritis. Commissioners and GPs reported that podiatry services were valued, however they were aware that podiatry services existed within a wider, more complex health and social care system, concern was raised that there was pressure to demonstrate the outcomes of cost reduction in services such as podiatry that existed between those systems with known variation within commissioning localities in evidencing service delivery and effectiveness.

“For me, everything has a spectrum and I think it’s about outcome, it’s not about service. So I don’t think for me, it’s less about what you buy; it’s more about the outcome that you’ve bought for the patient. So, in that instance, if the outcome was the patient needs to be able to walk to the shops, because it’s really important. And actually, that’s a big part of loneliness and isolation and that gets into that bit around actually, what’s the health and wellbeing objective for this person?” (Commissioner-code – 4).

“One of our providers is more cost effective than the other. Using their podiatrists, a lot more productively than the other.” (Commissioner – code-3).

One GP pointed out that it was often not an option to refer patients onto the treatment pathway to podiatry services.

“I will chat with them (patients who have arthritis) and not routinely refer due to the limited resource of podiatry.” (GP-code-1).

The difficulty of evidencing the impact of patient centred care was acknowledged by patients as well as the challenges in placing a monetary value on it. Patients empathised with healthcare workers and leaders that there would be challenges to running and managing services with the vast array of conditions, lower limb problems and health care needs of the populations they support. That said, patients were unsure if they would be able to definitely state that the money invested in podiatry services to manage foot care needs was cost effective.

“I don’t know if it’s good value for money. I wouldn’t say the relief was enormous. It is just more comfortable.” (Patient-code-SP4).

Within the patient focus groups there was a strong sense of guilt at wasting resources. Those who had access to a podiatrist who specialised in rheumatology were grateful and acknowledged this as a privilege over those who had not. In one focus group an emotional discussion took place between a patient who had RA and had access to podiatry and was very happy with the service and a patient who had OA and double hip and knee operations with continued limited mobility and was unable to access podiatry. However, the main areas of guilt centred around prescribed footwear and not being able to follow treatment plans given due to their disability. One patient stated.

“I would rather have more nail care appointments rather than shoes that don’t fit” (Patient-code -LP18).

### Theme three: understanding what podiatry services have to offer

This theme presents the ideas, thoughts and perceptions about the role of podiatry in modern health and social care services. Interestingly patients weren’t concerned with the health professionals’ job role per se, they just wanted someone to look at their feet and be offered options for access to an expert in podiatry/foot care services.

“… someone who was qualified. If you got somebody who is not, you might as well do it yourself.” (Patient-code -LP2).

Those who have had access to podiatry services and foot health management valued the impact of that care.

“I suppose it looks as though I’ve been very fortunate in the way I’ve been treated. I started in 1961 so it’s a long time and in that time I have seen consultants and when they felt I was ready for something to be done with my feet I was referred to podiatry and orthotics so I feel I’ve been well cared for even though the problem of making the shoes takes the time but in the end I get them but I do see having a one stop”. (Patient-code-LP5).

Commissioners and GPs know what skills podiatrists have and that they rely upon the podiatrists as leaders to voice the patient needs.

“They’re very skilled professionals in dealing with foot problems I suppose. But they cover a wide variety of things. I mean obviously there is the medical stuff, the diabetics and there’s the more mundane stuff, such as toenail care and things like that and surgery for ingrown toenails. And then there’s all the way through to management of fungus and toenail, toe deformities and management of things like toe fasciitis at the same time designing custom orthotics. So, the bio-mechanical problems, bio-mechanical assessments in patients …. that sort of thing”. (GP-code-1).

“I think they’ve [podiatrists] got the complicated stuff. And I’m not a podiatrist so I wouldn’t be able to say to you that’s that and that’s that, I rely on them to tell what they think is the most important things that they have to do”. (Commissioner-code-1).

On the other hand, patients were less likely to understand the diversity of skills that podiatrists have or the training standards met for service requirement to meet different levels of foot care needs.

“… well after listening to everybody today [talking about who they access for foot pain] I’m not so sure because I am confused in my own mind who does what, for what reason and what purpose. So, I would have to say I’m totally unclear.” (Patient-code LP13).

“… a lot of us don’t know the difference between the names of people have because before we would have seen a chiropodist, now chiropodists don’t exist but if you don’t know who you’re supposed to be seen for what is wrong with your feet, how do you even start. The terminology is very confusing”. Patient-code-LP7).

### Theme four: person factors of foot pain

This theme presents the reflections of people living with arthritis and their support network (carers, family, friends). Patients commented on the ability to find everyday tasks challenging and that others do not understand what they have to go through sometimes on a daily basis. They reported that their arthritis affected their mobility and that they hadn’t realised pain in their feet could be so severe. It was also highly reported that pain impacted on their mental well-being as well as their physical well-being.

“if you’ve got a problem with your feet, it’s awful if you can’t walk. There’s so much you can’t do.” (Patient-code SP3).

“If your feet don’t work or they are painful it impacts on the whole quality of your life from what you do recreationally, socially, work if you are still working. It impacts on everything. *NEW SPEAKER*: It’s a psychological impact not being able to wear a nice pair of shoes”. (Patient -code-LP6 &7).

Patients also commented on how arthritis is not always an easy condition and some patients commented that this condition might remain unseen by others who do not have it.

“If your feet don’t work or they are painful it impacts on the whole quality of your life from what you do recreationally, socially, work – if you are still working. It impacts on everything.” (Patient-code-SP1).

“Nobody understood because they can’t see it [foot pain/arthritis], the broken leg can be seen”. (Patient-code LP4).

“What you were saying, one thing that I don’t think people that don’t have arthritis realise is the pain you can be in. They don’t appreciate the excruciating pain you can have and how debilitating it and tiring it, if you’re taking tablets it can make you sleepy or woozy or upset stomachs. People that don’t have arthritis don’t realise what you have to go through sometimes on a daily basis … .. when I go to the golf course my friends think it’s funny I’ve got a pocket full of tablets … .they have got a headache I’ve got drugs. I never leave the house without pain killers. And I don’t think they appreciate the amount of pain they [people with arthritis] can be in. She’s a bit grumpy this morning; I can’t move I’ve got pain”. (Patient-code-SP3).

Commissioners and GPs were aware that people living with arthritis may not receive as much support in terms of footcare management compared to people living with diabetes. However, one participant explained that, in their defence, such services are not requested and highlighted the importance of people living with arthritis sharing their thoughts.

“The pathway service is monitored through the level of complaints … no complaints from people with arthritis … therefore the arthritis foot doesn’t get anywhere near as much support because we’re never asked to.” (Commissioner-code-1).

### Theme Five: facilitators of foot care

This theme captures Commissioners’ and GPs’ suggestions for the future shape of podiatry in modern healthcare to facilitate foot care. From Commissioners and GPs there was a strong emphasis on their use of NICE guidelines to steer their decision making through robust evidence to support the outcomes of cost reduction. They recommended that podiatrists get involved in the production of such guidelines and to work with them to meet both local and national needs for management of foot problems. However, one commissioner referred to the current status of health policy being funded for short term rather than long term producing a challenge towards achieving this, such that a proposal for a ‘new service’ has to include bridge funding for doing two things at the same time while the services are transfigured.

“We’re struggling to buy new things that are about lifesaving cancer drugs, let alone preventative healthcare. Even though it’s ridiculous because that’s where we should be investing, but when you’ve got the immediate problem in front of you for this year; it’s that burning platform issue isn’t it? I’ve got to put this fire out, I haven’t got a choice. But actually, it wouldn’t have even started if we’d have invested further up the pathway.” (Commissioner-code-2).

To solve this, among the commissioners and GPs there was a belief that a traditional clinical approach encourages dependencies and that podiatry services fell into the model of ‘paternalistic care’(a health care professional makes a decision(s) based on what he or she discerns to be in the patient’s best interests, even when the patient can make decisions for themselves). They expressed a need for change, calling for an increased focus in patient self-management and supported self-management for some foot problems to meet the foot care needs of patients within an evolving healthcare system. Yet, empowerment of patients to manage their own foot health needs across the UK as a facilitator of change has met with challenges from both clinicians and patients.

“it’s a big shock particularly for people who have had very paternal relationships with clinicians, you know, where, you know “tell me what to do, oh its lovely to see you again, how’s your mum” you know all this sort of thing, as opposed to actually, “Well, what did you do? How are you going to get out of this? What are your options? What do you think of these options I’m giving you?” (Commissioner-code-1)

They proposed stakeholders’ events and patient consultations led by the podiatry services as a mechanism for determining local population service needs.

“Everybody. We’ve had multiple stakeholder events, patients, carers, clinicians, all come together, some separately and sometimes together. Our partner organisations like Age UK … , we’ve run public forums where they’re talking next time about the acute service redesign, so everybody has an input. It takes it slower, but it gives people longer to think about things. We’ve been working on ‘my life, a full life’ now for … quite a few years.” (Commissioner-code-1).

## Discussion

Using focus groups and semi-structured interviews and a thematic approach to data analysis, this study has provided unique insights into stakeholder (patients’, commissioners’ and GPs’) perceptions of referral, access, provision and treatment for foot problems for individuals living with arthritis. Our overarching findings indicate that patients, commissioners and GPs have very similar experiences of limitations in referral, access and provision of treatment for foot problems for patients living with arthritis, yet by no means are these always negative. These findings build on our previous recommendations from our investigation of podiatrists’ experiences [21] and emphasise the need for a transformational approach in shaping UK podiatry services for people living with OA/RA. The resultant key themes constructed from our investigation are discussed below:

### Systems working together / navigation of care pathways

Podiatry / foot care services are found within the NHS health systems of acute, primary and social care as well as within the private sector [29]. Whilst this was perceived as a positive aspect within professional development by podiatrists in our earlier investigations, it appears to have created many different pathways that patients and commissioners find challenging to navigate. Within the United Kingdom, the gate-keeping role of GPs which has been accepted practice for over a century [9] was founded using a simple referral process. The driver for establishing a means of GP referral was the advent of medical specialisation during the last decades of the nineteenth century; this led to an initially informal system where GPs could refer patients to specialised colleagues, while still maintaining a continuing relationship with the patient [5, 7, 30]. This early feature – which required the presentation of a “visiting card” in order for the patient to be seen by a hospital physician, was primarily aimed at protecting the income of GPs [5, 31]. Subsequently, embraced as it was by the Dawson report [6], the system of referral to specialised services via GP letter became established. In contemporary healthcare, patients remain reliant on their GP referring them to an appropriate clinician at the right time, in the right place’ who is able to advise them and/or manage their foot health needs.

The Five-Year Forward View stresses new relationships with patients and communities via new care models and a modernised workforce to address chronic illness, to be co-ordinated around the patient’s needs, and, importantly, to “reduce variations in where patients receive care” (p8, 5-year forward view) [32]. The NHS Long Term Plan also stresses the need to “bring together different professionals to co-ordinate better care” (P 1 of summary document, NHS Long Term Plan), and “encourage more collaboration between GPs their teams and community services, as integrated care systems plan and deliver services which meet the needs of their communities [33].

### Finance / financial variations in services

As Table 3. (Themes and subthemes) summarises, respondents attributed the variation in podiatry /foot care services is attributed to the wider, more complex health and social care system yet commissioners in this investigation indicated that they are required to address these variations to primarily promote cost reduction, as opposed to development of new services.

During the early part of the twenty first century the dominant discourse turned to the need for a sustainable national economy, which profoundly influenced political-economic thinking and practices in healthcare provision [23]. After 2008, in the wake of the global financial crisis and embodied within the rhetoric of austerity, it became common to assert that the demise of the UK welfare state was inevitable [22]. Strategies to facilitate and legitimise service reduction or non-provision were couched in terms of service user empowerment, self-management [34–36] and patient activation [37]. Thus, the dominant narrative of austerity legitimised the non-provision of some NHS services for people with chronic conditions such as arthritis.

With echoes of how protecting GP’s income prompted the establishment of the referral system, [5, 31] in 2004, once more driven by a reduction in GPs’ remuneration [38], the BMA negotiated a pay-for-performance scheme with the government which became known as the Quality and Outcomes Framework. Under the terms of this, the largest health related pay-for-performance scheme in the world [38], provision of NHS foot health services saw a paradigm shift as the focus moved away from the management of foot pain and became screening and management of the foot related complications associated with diabetes [19, 39]. The unintended consequence of this change was to reduce the numbers of NHS podiatrists allocated to provide musculoskeletal services [21].

### Understanding what podiatry services have to offer

Previously we have reported that podiatrists expressed key concerns of frustration that, although podiatry has evolved as a profession, there remains a sense of misunderstanding, by non- podiatrists and patients, of the scope of practice and ability of podiatrists in what they do. This was similarly reported by most patients living with OA or RA, who also experienced confusion around what services are available to them, who are the gatekeepers to giving them the access they need to utilise foot health services and knowledge on what foot health services should they have now and in the future. Patients reported that most of their understanding has been found following an appointment with a GP, conversing with friends about their health or through self-directed directed learning to better understand their condition and prognosis. In contrast, almost all stakeholders, including the patients within this investigation acknowledged understanding of podiatry scope of practice, skills and training. As noted above, it is the pathways for referral and access to podiatry / foot health services that are the main concern of patients, commissioners and GPs.

This may be set to change. From April 2020, in the UK NHS, there will be GP contract changes, including extension of the Quality and Outcomes framework and enhancement of the “additional roles reimbursement scheme” - to encompass allied health professional (AHP) capability in primary care [40]. Recruitment of podiatry (as well as dietetics, occupational therapy, pharmacy technician, care co-ordination and health coaching) services will attract full reimbursement. In setting out the terms of the updated GP contract, the General Practitioners Committee England, NHS England and NHS Improvement are clear that all Primary Care Networks are expected to seek to utilise 100% of their available funding each year – and that the funds should only be spent on the additional roles specified in the document.

### Person factors of foot pain

Within all focus groups and interviews there was much debate over perceived split systems of podiatry, notably diabetes v musculoskeletal and that ‘arthritis is invisible’. People living with arthritis claimed that their arthritis was invisible, and that their foot health needs were, in the main, not being met. In contrast to this, commissioners and GPs suggested that this was because those patients were not sharing their thoughts with their GPs about their foot health needs. To that end, Commissioners saw themselves as ‘brokers’ to achieve more productive collaborations between GPs, podiatrists and patients. This though highlights an issue of whether service-users are well equipped to raise their needs in this way within the patient/health professional relationship - where power inequalities have previously been highlighted [41–43].

In the near future this may be compounded by the new Primary Care Networks who may choose to work with existing community-based partners, allocating the funds to joint or rotational posts; however as the tenor of the document is of employment of additional specified healthcare professionals within the Primary Care Networks, additional primary-care podiatry posts may be created. Interestingly the only AHPs to be allocated an agenda for change (AFC) banding of 7 - 8a are “first contact physiotherapists” [40]. This title is a departure from the previously agreed “first contact practitioners” introduced in the Musculoskeletal First Contact Practitioner Services document [44] which acknowledged that a range of healthcare professionals may meet the capabilities detailed in the Musculoskeletal Core Capabilities Framework [45], including podiatrists, osteopaths and occupational therapists. Within the updated GP contract [40] the indicative level for podiatry is at AFC band 7 and the specification is written to encompass musculoskeletal podiatry, core podiatry [46, 47], nail and soft tissue surgery and amputation-prevention podiatry. Thus, GPs may elect to contract or employ podiatrists who have maintained a broad scope of practice (including the musculoskeletal skill-set). Underpinned by their long-held rights to independently assess, diagnose, treat and discharge, such podiatrists are well placed to provide services in primary care settings. However, placed at AFC band 7 and in the face of a national shortage of podiatrists, it remains to be seen whether these posts attract podiatry applicants.

### Facilitators to foot care

Stakeholders within this investigation agree with podiatrists [21] that provision of podiatry/foot care services for individuals with arthritis is an area that continues to lack guidance. According to commissioners and GPs in this investigation, specific guidance (NICE guidelines) for foot health for individuals with arthritis conditions is essential as they use such guidelines to steer their commissioning decision making to support cost reduction. However, whilst podiatrists previously interviewed called for the reform of the current accessibility to services to one that matches the foot care needs of individual patients [21], there remains very little engagement of podiatrists as key members of National Guideline committees [20]. Podiatrists therefore need to take ownership of ‘foot care’ and produce and embed robust evidence within national and local guidelines [20, 46, 48].

Commissioners, GPs and patients in this investigation, in alignment with previously reported podiatrists’ views [21], were keen to explore alternative ways to promote podiatry services for procurement and new models of service provision, to be more reflective of people’s individual circumstances.

Suggestions of facilitators to this included larger stakeholder events to determine local foot health service needs and information that signposts a patient at their ‘first contact’ with their GP to help simplify the different aspects of podiatry / foot care services that are currently available and in which health system they are established.

### Strengths and potential limitations

This study examined the perceptions of patients in two regions of the UK, as a potentially representative snapshot. By using the experiences of two purposeful groups of patients from two disparate regions, rich text and themes have been generated.

Semi structured interviews with GPs and commissioners from the two zones provided the perspectives of those responsible for purchasing and for granting access to podiatry treatment on the behalf of patients. One respondent who was both a commissioner and a GP was informed by this dual role.

Limitations are acknowledged as both participant representative groups are in England, therefore data may not be wholly representative of the four home UK nations (England, Scotland, Wales and Northern Ireland) meaning proposed themes may be more or less significant in other areas. This may, however, align with high degrees of variation in specialist rheumatology service provision across the UK, wherein podiatry remains a notably poorly represented profession [49]. We also acknowledge that the GP and commissioner sample size was small and therefore may not completely represent the wider experiences of those stakeholders working in different regions of the UK. This group of stakeholders proved particularly challenging to recruit with the main reason for not participating being limited time available from their busy caseloads to participate. Some non-participants did however respond to our recruitment drive and acknowledged that, although they couldn’t participate, this was a worthwhile investigation of a neglected area.

Nevertheless, this paper provides a solid foundation from which researchers and clinicians can begin to understand how access and referral to podiatry / foot care services may be improved for individuals who have arthritis.

## Conclusion

The findings indicate that patients, commissioners and GPs have very similar experiences of referral, access and provision of treatment for foot problems for patients living with arthritis. Essentially, commissioners and GPs interviewed called for a transformational approach in current systems to include newer models of care that meets the foot care needs of individual patient circumstances. Patients interviewed called for better signposting and information of the different services available to help them manage their foot health needs. To address this, we have formulated a signposting pack, ‘the barometer of foot health needs©’, for all stakeholders to help them facilitate access to appropriate clinicians ‘at the right time, in the right place’ to manage foot health problems. The ‘Barometer of Foot Health Needs©’ is currently being tested for feasibility (ISRCTN registration: 10.1186/ISRCTN13564562).

## Data Availability

The anonymised data that support the findings of this study are available from the corresponding author upon reasonable request.

## Appendix

### Outline Questions / Structures for the Focus Groups and Interviews

The topic lists for the focus groups and interviews followed a semi-structured format. The following are examples of opening questions for each participant group to give a flavour of the broad areas of questioning. Openings arising from each question were followed up with further prompt questions.

### Patients

How do you access footcare or podiatry services?

Tell me about your experiences of accessing footcare services?

What information do you have on podiatry services and where do you get it from?

If you were the local Podiatry manager, how would you shape the service to your needs?

### GPs and Commissioners

How do you make a decision about commissioning foot care?

What guides your decision making on commissioning footcare services/ podiatry?

What information do you have on podiatry services and where do you get it from?

How do you differentiate commissioning of footcare services of different patient groups?

## Abbreviations

AFC: Agenda for change
ARMA: Arthritis and musculoskeletal alliance
CCG: Clinical commissioning group
CIRC: Clinical innovation research centre
CPRD: Clinical practice research datalink
GP: General practitioner
NHS: National health service
NICE: National institute for health and care excellence
NIHR: National institute for health research
OA: Osteoarthritis
PPI: Patient public involvement
RA: Rheumatoid arthritis
UK: United Kingdom
